# Commentary: Push–button analyses

**DOI:** 10.1155/S1463924680000375

**Published:** 1980


### Commentary

### Push-button analyses

While attending the recent "Pittsburgh Conference" and hearing the many comments and discussions about the automation of chemical analyses, I was reminded of an address entitled *Push-Button Analyses* that I presented years ago (American Chemical Society Award address in Instrumentation, Los Angeles, California, Spring 1963). So a few days ago I dug it out, dusted it off, and realised that many of my thoughts and concerns about the automation of chemical analyses seventeen years ago are still pertinent and important today. Therefore, I would like to quote portions of that 1963 address and close with a few comments from a 1980 perspective.

". . . One of last year's ACS award winners (1962), Dr Herman Liebhafsky, in presenting his award address and in referring to the future of analysts, stated, 'determinators are more likely to push buttons and handle chart paper than to read burettes, and they will yield ground to maintenance men as automation increases'.

"The first part of this statement could be considered symbolically or literally. For my purposes, I would like to assume that he meant a literal interpretation. In other words, rather than consider that the statement implies the abandonment of the burette, I would like to suggest that the burette and some other classical laboratory devices be brought up-to-date and used as building blocks to automate various analytical procedures. The quantitative controlled delivery of reagent will be a necessary laboratory operation for many years, but in place of stretching the neck and straining the eye to read the meniscus in a burette, it should, of course, become common practice to push buttons and read the volume off a chart, or what is often more desirable, to obtain a digital readout or printout of volume.

### Building blocks for automation

"The second part of Dr Liebhafsky's statement implies evolution towards an automated laboratory where we will not stop to read volumes, if indeed reagents are still quantitatively delivered in the future, but instead all data will be automatically handled, evaluated and acted upon. But automation is a concept rather than a goal. And I would rather talk to you about a relatively simple and presently practical approach to automating a few quantitative analytical methods than to speculate too much about the automatic wonders of the future. Still, it is good to dream so I will conclude this talk with some ideas about the push-button chemical laboratory of the future and the implications with regard to chemical training.

"Right now, it it is back to the burette, the pipette, and the filter. These devices are necessary building blocks for an approach we have often used in automating various reaction-rate, titration, and null-point systems. However, in order to automate these methods, it is important to shift from the classical designs of burettes, pipettes, filters, etc to new push-button versions.

". . . This system illustrates a building-block concept of automation which is versatile and decreases the problem of obsolescence. Each one of the units is a laboratory device of general usefulness . . .

". . . With these few examples, I hope it is apparent that a versatile building-block system could be a practical way for many laboratories, including research laboratories, to at least partially automate without running the danger of rapid obsolescence. Also, it has obvious advantages as an educational system. One argument for laboratory automation is certainly the possibility of freeing scientists from being used as control systems so that they will have more time for creative efforts. With the present shortage of scientists, this is certainly an important aspect. However, more and more automation will probably be introduced for an another reason: often, people just do not fit smoothly into a complex organisation of instruments, at least not without exhaustive training such as with our astronauts. Unfamiliarity with instrument characteristics can cause operational and maintenance problems, and the all-too human mistakes and contaminations can cause serious errors. The use of human series participation (such as with a series of chemical service laboratories working separately on a single problem) can lead to inefficiencies and too many mistakes.

### Change of emphasis

"So, the emphasis will eventually shift to an integrated organisation of automatic analytical instruments controlled by instrument servos instead of the present practice of using human servos, often in a haphazard way. Such a changeover seems feasible when one compares the human servo response to a command with that of an instrument servo. In the case of the human, a command, for example an instruction to move an object to a given location, is picked up by the eye, ear, or touch, and a signal is transmitted to the human brain, which controls the body muscles that exert a force on the object to move it to the desired location. The eye or other sense acts as an information-feedback device that describes the actual position. The mind compares the command and feedback information and controls the body so as to change the load position in accordance with any error signal – i.e. the difference between desired and actual positions of the object. The man-made servo operates in a similar way. The command v is picked up by a device that puts it in the form of an acceptable command signal r, and the information on the actual position of the load is put in the form of a feedback signal b. These are usually voltage signals that can be compared to give a difference voltage e that is fed to an electronic signal controller. The controller uses the error signal e to regulate the electrical power to the motor (or other positioner), and the motor operates to move the load to the desired position. When the load is at the desired position, the error (actuating) signal e is essentially zero. At present, the instrument servos do not have the versatility of the human, but they can be made to perform many specific tasks more precisely, reliably and rapidly than humans, and to perform many tasks that are just humanly impossible.

"However, with a complex analytical operation it is staggering to think of the automated monster that would be created by combining present-day instruments into one completely automatic analytical system. Therefore, only through some elegant developments that make maximum utility out of a minimum amount of equipment will it be feasible for full automation to become economically and technically practical in laboratories with a wide variety of analytical problems. This is a challenge for the future.

"Perhaps, if we aspire to full automation we should add a new statement, i.e. the maintenance men will then yield ground to automatic correction, repair and replacement – a sort of automatic rejuvenation process within the instrument itself.

"For future chemists to be prepared to make the most of instrument developments and eventually to be masters of robot laboratories, and merely to exist in a highly automated environment, it will be necessary to provide suitable training in the colleges and universities. A course in instrumental methods is now a chemistry requirement. But, I believe that something more is needed. Every chemist, in fact, every scientist would benefit greatly by some training in instrumentation as such – training that would provide a basic working ability and understanding of the electronic, mechanical, optical, hydraulic and other components that make up instruments. The need was well expressed in a recent letter to me from a chemistry professor who wrote, 'I am becoming concerned with my lack of practical knowledge in electronics – a fact that leaves me rather at the mercy of my instruments. Certainly for the best creative efforts, I should be the master of my instruments'.

"In an era of space platforms, trips to the moon and automated factories, I believe it will be imperative that all scientists obtain some perspective and understanding and preferably a working knowledge of the devices that provide their data. This would seem to be the scientific way. It is both practical and fitting that chemists prepare for a laboratory revolution or evolution that will provide the data for scientific achievements even greater than in the past and at an ever increasing rate. However, as it has been said, 'Things won't change completely in an automated laboratory, the button that gets ahead will still be the one with the most push'."

In this year of 1980, we have a wide choice of elegant and versatile commercial push-button titrators, burettes, pipettes, reaction-rate analysers, and a host of other automated instruments. There is more and more talk and a few examples of automated laboratories, automated research, automated calibration and repair. However, there is still a general lack of good scientific instrumentation/automation courses in colleges and universities. It would seem that the time has come for a vast improvement in this important area.

*H. V. Malmstadt*

### Forthcoming articles in the Journal of Automatic Chemistry

The following papers are being considered for publication in *The Journal of Automatic Chemistry:–*

Improved accuracy in automated chemistry through the use of reference materials.

The flexible dialyser membrane – a source of error in plasma creatinine determination.

Simple method for calculating frequency of standardisation.

Amperometric systems for the determination of oxidase enzyme dependent reactions by continuous flow and flow injection analysis.

Multilevei analysis of variance used to partition components of optical imprecision in an analyser system with disposable cuvettes.

